# Advancing solar energy applications with graphene: the potential of minimally oxidized graphene

**DOI:** 10.1186/s40580-025-00498-x

**Published:** 2025-06-27

**Authors:** Qiang Chen, Jewook Kim, Myungwoo Choi, Seokwoo Jeon

**Affiliations:** 1 Department of Materials Science and Engineering, Korea Advanced Institute of Science and Technology (KAIST), Daejeon, 34141 Republic of Korea; 2 Department of Materials Science and Engineering, Korea University, Seoul, 02841 Republic of Korea; 3 Nano Hybrid Technology Research Center, Korea Electrotechnology Research Institute (KERI), Changwon, 51543 Republic of Korea

**Keywords:** Minimally oxidized graphene, Non-oxidized graphene flakes, Graphene quantum Dots, Solar energy conversion, Photovoltaics, Photothermal conversion, Photocatalysis

## Abstract

Integrating carbon nanomaterials into solar energy technologies has emerged as a promising strategy to improve efficiency, scalability, and sustainability. Although graphene has excellent carrier mobility, electrical conductivity, and optical transparency, graphene derivatives such as graphene oxide (GO) and reduced graphene oxide (rGO) suffer from significant structural defects and disruption of the sp^2^-hybridized carbon lattice caused by oxidative processing, severely limiting their electronic and optoelectronic performances. To address these limitations, minimally oxidized graphene (MOG), which includes non-oxidized graphene flakes (NOGFs) and low-oxidized graphene quantum dots (GQDs), has been developed via a nondestructive approach based on ion or molecular intercalation followed by liquid-phase exfoliation. These materials retain the integrity of a π-conjugated network and offer tunable functionalities and solution processability. NOGFs exhibit high conductivity, broadband light absorption, and thermal stability, making them ideal materials for use in solar cell electrodes, photothermal absorbers, and photocatalytic scaffolds. GQDs with tunable bandgaps and abundant functional groups serve as interfacial modifiers in solar cells and as active sites for photocatalysis. This review summarizes recent advances in MOG, focusing on structure–property–performance relationships and applications in solar energy conversion. A comparative evaluation with conventional GO/rGO-based systems is presented along with future directions toward developing high-efficiency graphene-enabled solar technologies.

## Graphical Abstract

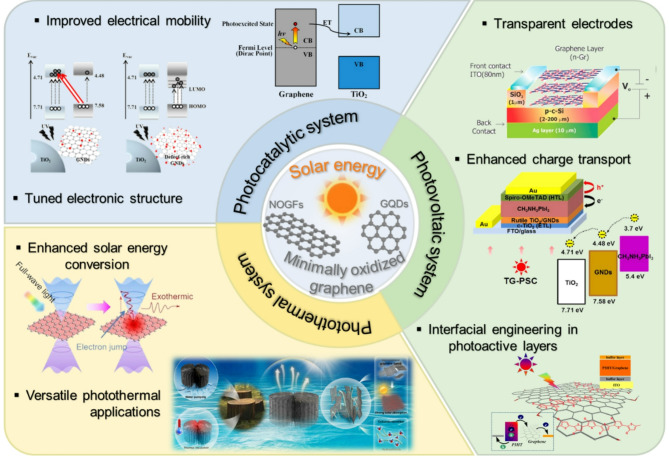

## Introduction

The growing global demand for clean and sustainable energy has accelerated advancements in solar energy technologies, making solar energy an essential component for future energy systems [1]. In 2022, photovoltaics accounted for over 50% of global power capacity additions, achieving substantial cost reductions and improved scalability [2]. Although conventional materials such as Si-based photovoltaics [3], plasmonic metal absorbers for photothermal conversion [4], and metal oxide/sulfide photocatalysts achieved significant advances in terms of solar energy utilization [5], they share some common critical limitations, including light-induced degradation, low quantum efficiency, poor mechanical flexibility, and limited tunability of electronic or optical properties. These intrinsic constraints have driven the search for advanced materials that can simultaneously offer structural versatility and enhanced optoelectronic performance.

To this end, two-dimensional (2D) materials have emerged as promising candidates for various solar energy conversion systems [6, 7]. Among these materials, graphene is particularly attractive because of its exceptional intrinsic properties such as ultrahigh carrier mobility, broadband light absorption, high thermal conductivity, and mechanical flexibility, which makes it a highly versatile platform for diverse solar energy applications such as photovoltaics, photothermal systems, and solar-driven photocatalysis [8, 9]. Despite the exceptional intrinsic properties of graphene, its practical integration into solar energy devices is limited because of challenges associated with the large-scale production of high-quality sheets. To address this issue, graphene oxide (GO) and reduced graphene oxide (rGO) have been widely explored as accessible alternatives given their ease of synthesis, tunable surface functionalities, and solution processability [10, 11]. These materials have been successfully used in diverse solar applications. In photovoltaic systems, Lin et al. reported that inserting a 2-nm-thick GO interlayer between the active layer and indium tin oxide (ITO) electrode in polymer solar cells increased power conversion efficiency (PCE) from ~ 1.8% (ITO only) to ~ 3.5%, which was comparable to that of conventional PEDOT: PSS-based devices [12]. Jokar et al. employed GO and sulfonated rGO as a hole-transport layer in perovskite solar cells, achieving a PCE of 13.8% with GO and over 16% with rGO, along with good mechanical flexibility and reduced charge recombination [13]. For photothermal applications, Li et al. fabricated rGO-based aerogels combining sodium alginate and carbon nanotubes, achieving broadband solar absorption (~ 92%) and a water evaporation rate of 1.62 kg m^− 2^ h^− 1^ under one-sun illumination, with a conversion efficiency of approximately 83% [14]. For photocatalysis, Hsu et al. demonstrated that GO could act as a visible-light-responsive photocatalyst for CO_2_ reduction, achieving methanol production rates approximately six times higher than pristine TiO_2_ [15]. Yang et al. incorporated GO into a MIL-68(In)-NH_2_ framework, where its presence significantly enhanced visible-light-driven antibiotic degradation by promoting charge separation and light absorption, resulting in over 90% pollutant removal within 210 min [16]. Although these examples highlight the functional versatility of GO and rGO for solar applications, critical limitations persist. The partial restoration of a sp^2^-hybridized carbon lattice network during the chemical or thermal reduction of GO results in residual oxygen defects and structural disorder. These defects reduce electrical conductivity, which is critical for charge transport in photovoltaic devices; introduce recombination centers that impair photocatalytic performance; and lead to instability and aggregation during prolonged photothermal operation [17]. Therefore, the precise control of the oxidation level, structural integrity, and defect density of graphene is crucial for achieving optimal balance between conductivity, stability, and functionality. This requirement highlights the increasing importance of graphene with less defects as a next-generation material that can overcome the inherent limitations of conventional GO and rGO, enabling high-performance solar energy conversion technologies.

To overcome the limitations associated with conventional GO and rGO, minimally oxidized graphene (MOG), particularly non-oxidized graphene flakes (NOGFs) and low-oxidized graphene quantum dots (GQDs), have emerged as promising candidates for advanced solar energy conversion. The NOGFs and GQDs are fabricated through the controlled exfoliation of graphite intercalation compounds (GICs) using mild, minimally oxidized intercalation-exfoliation methods. For example, NOGFs are produced by intercalating graphite with ions or molecules followed by gentle solvent-assisted exfoliation, yielding large flakes with minimal oxidation ( < ~ 5 at % oxygen content) and low structural defects (ID/IG ratio of Raman peaks < 0.065) [18–23]. In parallel, low-oxidized GQDs (~ 5 at % oxygen content, much lower than ~ 20 at % in conventional GQDs) can be fabricated by solvothermal exfoliation using potassium sodium tartrate as an intercalating agent and a solvent, followed by exfoliation in water [24, 25]. This approach yields highly crystalline GQDs with well-defined subdomain structures and discrete bandgaps (~ 3.1 eV) suitable for optoelectronic applications [26, 27].

These MOG preserve the intrinsic sp^2^-conjugated carbon lattice, which endows them with superior properties, including high electrical conductivity (> 3000 S cm^− 1^ for vacuum-filtered NOGFs film) [28], excellent thermal conductivity (~ 1.53 W m^− 1^K^− 1^ for NOGFs/epoxy composites with 10 wt % addition) [29], superior mechanical strength (over 230% improvement in toughness compared to pure epoxy, reaching 2.52 MJ m^− 3^) [30, 31], and strong broadband solar absorption (> 98% across 250–2500 nm) [32]. Low-oxidized GQDs demonstrate intrinsic deep blue-range photoluminescence (~ 400 nm) and tunable optoelectronic behaviors [33–37]. These remarkable properties can be attributed to their controlled oxidation state and preserved graphene structure, and they enable NOGFs and low-oxidized GQDs to overcome the inherent limitations of GO and rGO, making them ideal candidates for advanced solar energy conversion applications such as in photovoltaics, photothermal conversion, and photocatalytic systems [38]. The strong structure–property correlation observed in these systems underscores the critical importance of precisely managing graphene oxidation levels to maximize their potential in high-efficiency, multifunctional solar devices.

**Fig. 1:**
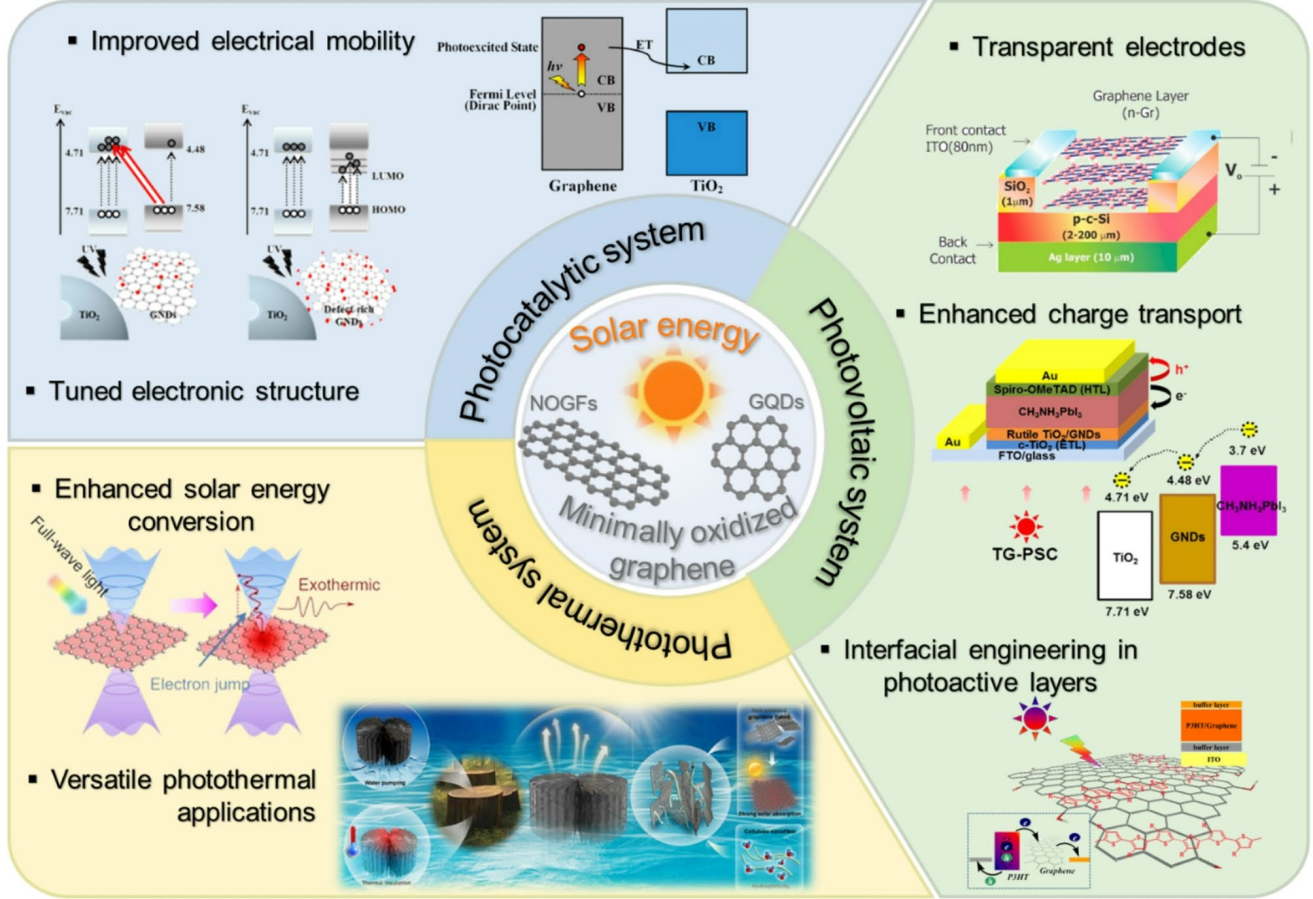
Schematic of the application of oxidation-controlled graphene in versatile solar energy technology including photovoltaics, photothermal, and photocatalytic systems. Reprinted with permission from Ref [32, 39–43]

This review presents a comprehensive and critical overview of MOG-specific NOGFs and low-oxidized GQDs and their emerging roles in solar energy technologies (Fig. 1). To this end, we systematically discuss synthesis strategies, structural features, and physicochemical properties of both NOGFs and low-oxidized GQDs, highlighting how variations in the oxidation level, domain size, and edge structure affect their optoelectronic behavior and functional performance. Further, we focus on the mechanism by which NOGFs and low-oxidized GQDs interact with light, charge carriers, and surrounding media, including their roles in photon absorption, charge transport, hot-carrier generation, and interfacial charge separation. Further, we explore the integration of NOGFs and low-oxidized GQDs into three major solar energy platforms (i.e., photovoltaic, photothermal conversion, and photocatalyst systems), highlighting how the preservation of the sp^2^ carbon lattice and minimization of oxidative defects enable enhanced device performance. Thus far, NOGFs have been discussed in the context of transparent electrodes, active absorbers in photothermal evaporators, and charge transport layers in photocatalysts, wherein their high conductivity and structural integrity facilitate efficient light-to-electric conversion, rapid thermal localization, and robust interfacial electron transfer. Conversely, low-oxidized GQDs have been evaluated for their tunable band gaps, strong visible and UV absorption, and abundant edge sites, which enable efficient light harvesting, exciton dissociation, and generation of reactive species under solar irradiation. We examined key design principles for next-generation graphene engineering, including oxidation-tuned electronic modulation, edge-functional group control, and scalable nondestructive exfoliation by comparing MOG with conventional GO/rGO systems. Furthermore, we identify ongoing challenges in synthesis reproducibility, stability, and device-level integration, and propose future research directions. This review aims to bridge the gap between fundamental carbon nanomaterial development and application-driven solar energy engineering. We position MOG as a distinct and versatile class of functional materials and offer new insights into how rational molecular-level designs can unlock their full potential for real-world, high-efficiency, and sustainable solar technologies. We hope to inspire continued innovation at the intersection of materials science, nanotechnology, and renewable energy.

## Fundamentals of MOG

### Fabrication strategies

MOG materials are synthesized via non-destructive or mildly oxidative methods that preserve the sp²-hybridized carbon lattice. These materials are structurally distinct from GO/rGO, which are typically produced through harsh oxidation-reduction processes that introduce significant defects and disrupt π-conjugation. They also differ from graphene synthesized by chemical vapor deposition (CVD), which offers high crystallinity but requires high-temperature growth and transfer steps, limiting its scalability and compatibility with solution processing. By using controlled intercalation and gentle exfoliation routes, NOGFs and GQDs combine high structural integrity with tunable surface functionality, making them ideal for scalable applications in optoelectronics and energy conversion [25, 38].

Initially, Kwon et al. employed thermal treatment of graphite powders with potassium salts such as KCl or KOH, followed by mild sonication in polar solvents [20]. This approach enabled exfoliation without oxidative damage, producing NOGFs with significantly reduced defect densities (ID/IG ≈ 0.1). However, the produced flakes typically had relatively small sizes (< 1 μm), limiting their practical applicability. A significant improvement came with Park et al., who introduced a low-temperature eutectic salt system consisting of KCl-NaCl-ZnCl_2_ [22]. This system allowed efficient potassium intercalation at notably lower temperatures (~ 350 °C), reducing structural damage. Subsequent sonication in pyridine enabled more uniform exfoliation, yielding larger and thinner graphene flakes with an average oxygen content as low as ~ 2.9 at%. Notably, films derived from these flakes demonstrated excellent electrical performance, achieving a sheet resistance of around 930 Ω sq^− 1^ at 75% transmittance and conductivity reaching up to 91,000 S/m, significantly outperforming earlier methods.

Advancing further, Kim et al. explored solvothermal intercalation techniques using quinoline as the intercalant [18]. Quinoline’s conjugated structure provided effective non-covalent functionalization, which not only facilitated the exfoliation process but also significantly improved dispersion stability in various solvents. The resulting graphene flakes exhibited increased lateral sizes (~ 4 μm), moderate thickness (3–10 layers), and stable dispersion capability, making them highly suitable for composite and barrier applications.

Most recently, Kim et al. introduced a novel ternary graphite intercalation compound (t-GIC) method utilizing KC_24_(THF)_2_, combined with spontaneous solvent insertion in highly polar aprotic solvents such as DMSO [19]. This strategy markedly reduced exfoliation energies through ion-dipole interactions, resulting in exceptionally large graphene flakes exceeding 30 μm (average lateral size ~ 30 μm), with ultra-low defect ratios (ID/IG ≈ 0.06) and significantly enhanced electrical conductivity (up to 3.36 × 10⁵ S m^− 1^ for fabricated film). The addition of polyvinylpyrrolidone (PVP) as a surfactant further improved the dispersion and stability of these graphene flakes, enhancing their practical applicability in various technological fields.

Extending these intercalation-based strategies, Song et al. developed a solvothermal approach to synthesize low-oxidized GQDs with controlled structural integrity [24]. Specifically, potassium sodium tartrate acted simultaneously as an intercalant and solvothermal medium, mixed with graphite at a mass ratio of 10:1, and heated at 250 °C for 24 h. The subsequent rapid exfoliation in water allowed the formation of high-quality GQDs with minimal oxidation. The resultant GQDs demonstrated narrow size distributions predominantly between 1 and 5 nm, thickness around 1 nm, and high crystallinity with a distinct lattice spacing of ~ 0.21 nm. Critically, this synthesis method significantly minimized oxidation, as confirmed by X-ray photoelectron spectroscopy (XPS), which indicated dominant intrinsic sp^2^ domains and negligible oxygen functionalities compared to conventionally produced GO-based quantum dots. Consequently, these low-oxidized GQDs exhibited strong photoluminescence emission around 400 nm and quantum yields up to 4%, enhancing their suitability for advanced optoelectronic applications such as light-emitting diodes (LEDs), which achieved luminance exceeding 1000 cd m^− 2^.

Overall, the development of NOGFs and low-oxidized GQDs synthesis strategies represents a substantial progression toward obtaining graphene materials with tailored structural, electrical, and optical properties, critical for broadening their application scope in diverse technological fields.

### Key properties and comparison with go/rgo

MOG materials exhibit superior structural, electronic, and optical properties owing to their preserved sp^2^-carbon lattice and low defect density. NOGFs retain near-pristine honeycomb structures, demonstrated by their exceptionally low ID/IG ratios (< 0.05) in Raman spectra [18, 22], indicating minimal defects, and high-resolution TEM observations confirming basal-plane continuity with vacancy defects below 0.1% [19]. Low-oxidized GQDs similarly maintain crystalline subdomains with a characteristic lattice spacing of 0.21 nm [24], and their edge functionalization enables solubility without disrupting the core conjugation [25, 37]. In contrast, GO exhibits significantly higher defect density, reflected by ID/IG ratios exceeding 1.0 and oxygen content ranging from 30 to 40 atomic percent [17], while rGO retains residual defects even after reduction, typically showing ID/IG ​values between 0.8 and 1.2 [10]. Electronically, NOGFs films achieve high electrical conductivities exceeding 3000 S cm^− 1^ [28], approaching the values for mechanically exfoliated graphene (~ 5000 S cm^− 1^) and greatly surpassing rGO, whose conductivity typically ranges between 10 and 100 S cm^− 1^ [22]. Low-oxidized GQDs, meanwhile, exhibit semiconductor behavior with adjustable bandgaps (2.5–3.5 eV), providing significant optoelectronic tunability that pristine graphene inherently lacks [33, 37]. Optically, NOGFs demonstrate exceptional broadband absorption (> 98%) across the solar spectrum (250–2500 nm), notably outperforming rGO (85–92%) [14] and GO (< 80%) [44]. Furthermore, GQDs display quantum-confined photoluminescence with emission peaks around 400 nm and substantial quantum yields of 4–10% [24, 37], significantly exceeding GO-based quantum dots, which typically exhibit quantum yields below 1% due to oxidation-induced quenching [45]. Additionally, NOGFs demonstrate superior thermal conductivity in composites, achieving approximately 1.53 W m^− 1^ K^− 1^ at a 10 wt% loading, roughly three times that observed in rGO composites, owing to their intact phonon transport pathways [29]. Mechanically, NOGFs-reinforced polymers display a toughness increase of approximately 230% relative to pure epoxy [30], whereas GO composites exhibit substantial degradation under thermal stress conditions [17]. A comparison of key properties among GO, rGO, pristine graphene, NOGFs, and low-oxidized GQDs is provided in Table 1.

**Table 1:** Comparison of key properties among GO, rGO, pristine graphene, nogfs, and low-oxidized GQDs

Properties	GO	rGO	graphene	NOGFs	Low-oxidized GQDs
Oxygen content (at %)	30–40 [17]	10–20 [10]	< 1 [55]	< 5 [19]	~ 5 [24]
ID/IG	> 1.0 [17]	0.8–1.2 [10]	0.05–0.1 [55]	< 0.065 [22]	0.1–0.3 [37]
Conductivity	< 0.1 [10]	10–100 [13]	> 10^3^ [43]	> 3000 [28]	N/A
Bandgap	insulator	Variable	zero	zero	2.5-3.5 eV [17]
Solar absorption	< 80 [83]	85–92 [14]	> 97 [32]	> 98 [32]	UV-selective [61]

## Applications in solar energy technology

### Photovoltaic systems

#### Mechanism of photovoltaics and advantages of MOG

Among various solar energy conversion technologies, photovoltaic devices can directly transform sunlight into electricity, offering scalability and long-term viability. However, conventional photovoltaic materials suffer from critical limitations such as insufficient light absorption, inefficient charge transport, and rapid recombination rates, which collectively inhibits their PCE and practical applications [46]. These limitations have motivated the development of nanomaterials with tailored optoelectronic properties, which have emerged as promising candidates for next-generation photovoltaic systems [47–49]. Graphene, which is a 2D monolayer of sp²-hybridized carbon atoms arranged in a honeycomb lattice, has attracted considerable attention because of its exceptional electronic conductivity [50–52], broadband optical absorption [53, 54], mechanical strength [55, 56], and chemical stability [57]. These unique features render graphene highly versatile across different layers of photovoltaic architectures, including transparent conductive electrodes (TCEs), charge transport layers, and active light-absorbing materials. The π-conjugated electronic structure of graphene enables efficient interaction with a wide spectral range of sunlight [58], from UV to near-infrared (NIR), thereby facilitating ultrafast charge carrier mobility (~ 200,000 cm^2^ V^− 1^s^− 1^) [59]. This enables graphene to serve as an efficient charge transport layer, suppress carrier recombination, and promote charge extraction in photovoltaic devices. However, the gapless structure of graphene limits its ability to generate a photovoltage [60], prompting the exploration of functionalized or modified graphene derivatives with tunable band structures.

GO is a heavily oxidized form of graphene incorporating diverse oxygen-containing groups such as hydroxyl, epoxy, and carboxyl groups, which improves its dispersibility and interface compatibility. In addition, PV studies have utilized its graphitic properties such as simple processability and high scalability. However, most of these studies are limited to using GO as a composite-based pillar in PV components, leveraging its good interfacial interactions and ease of chemical modification. This limitation arises from the disrupted π-conjugation network of GO, severely decreasing electrical conductivity and electron mobility. To overcome this problem, the reduction of GO to remove oxygen-containing groups and enhance its electrical properties has attracted attention for PV device applications [61]. These rGO-based studies demonstrate an improved PV performance through a restored gapless structure, and there have been cases reporting its application in TCEs. However, rGO does not exhibit sufficiently restored crystallinity, resulting in relatively poor conductivity and carrier transport, which limits its effectiveness as an electrode material.

In comparison, MOG offers a strategic balance by sufficiently preserving the π-conjugated framework during the fabrication process and retaining moderate functional groups to enhance interfacial interactions with other materials [19, 62]. Further, PV devices utilizing MOG have shown superior performance compared with those using GO and rGO; in particular [63], MOG-based TCEs demonstrated significantly enhanced performance compared with those based on rGO [64]. In addition, GQDs, which are nano-sized derivatives of high-quality graphene, can serve as perfect alternatives to GO [65–67]. These nanostructures exhibit discrete energy levels, tunable optical bandgaps, and improved photoluminescence compared with GO, which enables their use as active absorbers or energy-level modifiers in various solar cell platforms, including perovskite [39, 68], dye-sensitized [69], and organic photovoltaics [70, 71]. GQDs synthesized using oxidation-controlled top-down strategies exhibit enhanced electron mobility and stable energy-level alignment, which contribute to efficient exciton dissociation and charge extraction [24, 72, 73].

In conclusion, the rational design and integration of MOG highlight an approach for overcoming the intrinsic limitations of conventional photovoltaic systems. Simultaneously achieving high optical absorption, efficient charge carrier dynamics, and stable interfacial engineering is possible by tuning the oxidation state of graphene and preserving key aspects of its electronic structure, thereby pioneering new pathways for high-performance, scalable, and sustainable solar energy conversion technologies.

#### Performance comparison of graphene-based solar cells

**Fig. 2:**
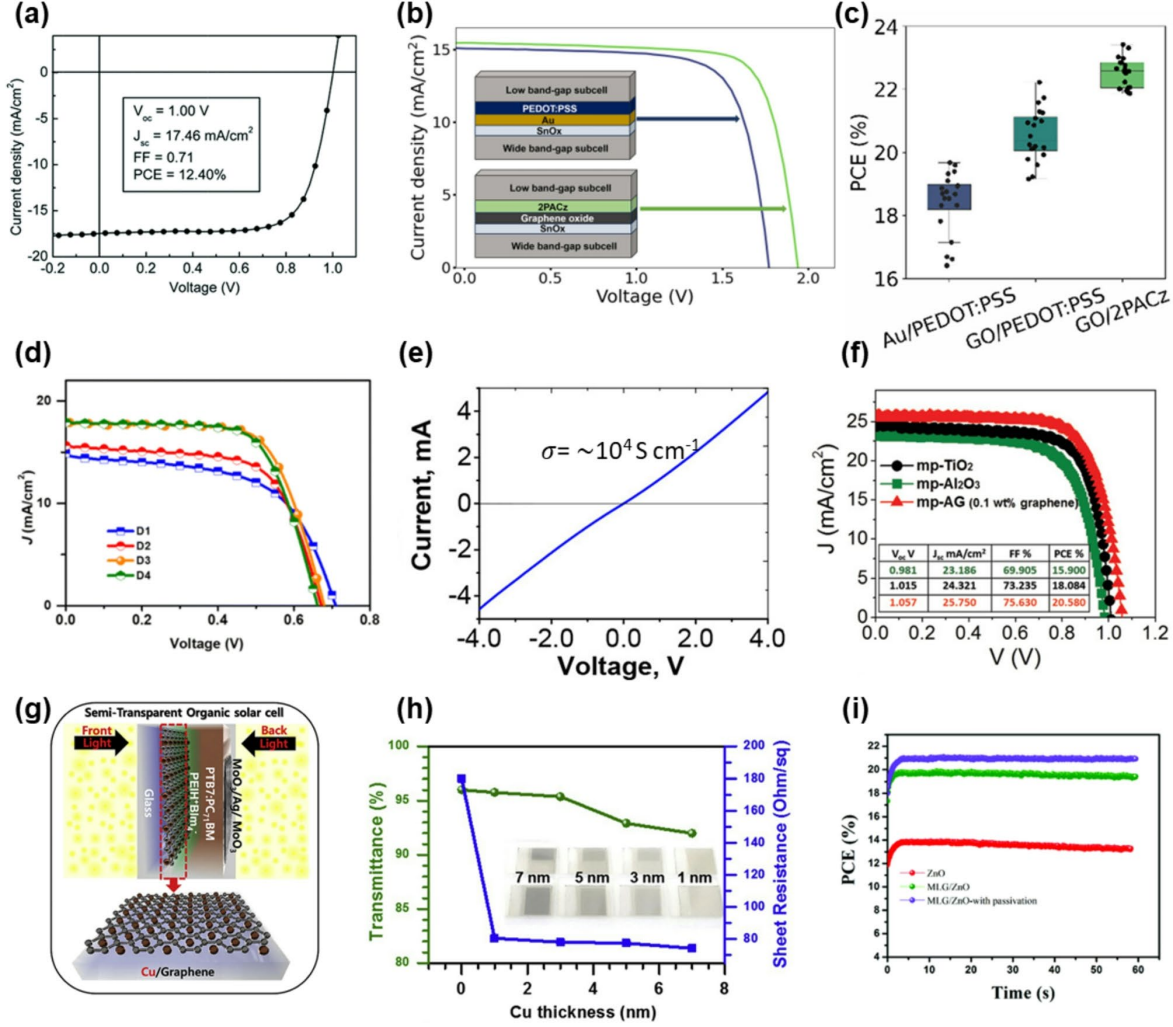
Comparison of GO, rGO, and MOG in a PV device. (**a**) J–V curve of fabricated planar heterojunction PSCs using GO as a hole conductor. Reproduced with permission from [74] (**b**) J–V curve of fabricated perovskite tandem cells using GO as an interconnecting layer. (**c**) PCE comparison of the fabricated perovskite tandem cells. (**b-c**) Reproduced with permission from [75] (**d**) J–V curve of fabricated DSSCs using rGO as an interfacial layer. (**e**) C–V curve of rGO-based TCE. (**d-e**) Reprinted with permission from Ref [76] (**f**) J–V curve of fabricated PSCs using rGO as a component of ETL. Reprinted with permission from Ref [77] (**g**) Schematic of organic solar cell using Cu/CVD-graphene as TCE. (**h**) Transmittance and sheet resistance of Cu/graphene TCE. (**g-h**) Reprinted with permission from Ref [64] (**i**) PCE comparison of fabricated PSCs using ZnO-based ETL. Reprinted with permission from Ref [63]

GO is widely utilized in solar-cell research because of its electrical properties and processing advantages. A planar heterojunction (PHJ) solar cell using GO as the hole conductor is fabricated as shown in Fig. 2a and exhibits a stable J–V curve [74]. The best device exhibits a V_oc_ of 1.00 V, J_sc_ of 17.46 mA cm^− 2^, and fill factor (FF) of 0.71, achieving a PCE of 12.40%. The rationally calculated J_sc_ value is consistent with the corresponding J_sc_ values obtained from J–V curves. This result can be attributed to the GO layer efficiently extracting holes from the perovskite, which facilitates the formation of homogeneous and large domains and improves surface coverage. Recently, GO has been used as a recombination layer of the interconnecting layer in all-perovskite tandem solar cells, which makes the 2PACz HTL a powerful candidate for replacing PEDOT: PSS (Fig. 2b) [75]. This device architecture results in significantly reduced optical and nonradiative losses, leading to a champion device efficiency of 23.4% compared to 19.7% with conventional layers (Fig. 2c). The introduction of GO to replace the Au recombination layer facilitates the use of a 2PACz HTL as an alternative to PEDOT: PSS, which contributes to both enhanced performance and improved stability compared to the reference Au/PEDOT: PSS structure.

The introduction of GO as a component of solar cells enhances PV performance; however, it is still difficult to consider this a remarkable improvement because of the intrinsically low electrical conductivity and high sheet resistance, both of which originate from the damaged crystal structure. Consequently, some studies attempted to incorporate rGO to achieve greater performance enhancement. For example, Bhand et al. chemically modified the FTO/TiO_2_ interface of dye-sensitized solar cells (DSSCs) by inserting self-healing rGO. The fabricated DSSC devices (D1, D2, D3, and D4) were classified based on the application of the rGO spin-coating treatment: D1 and D2 without treatment, and D3 and D4 with treatment. The PCE measured for D1 and D3 were 6.08 and 8.08%, respectively (Fig. 2d) [76]. EIS measurements for DSSCs indicate that improvement in the PCE can be attributed to the increased electron transfer and suppressed charge recombination at the modified FTO/TiO_2_ interface while maintaining the optical transparency of the photoanode caused by an intrinsically high transparency of rGO. Although GO has been widely employed in various photovoltaic applications, its application in TCE remains challenging. As shown in Fig. 2e, GO is converted into rGO films after thermal reduction. The rGO films can be employed as TCEs in photovoltaic devices and exhibit a high electrical conductivity of up to 104 S cm^–1^ and optical transparency of ~ 90%. Mahmoudi et al. demonstrated highly efficient and stable perovskite solar cells (PSCs) by incorporating a perovskite/Ag-rGO composite in the active layer and a mesoporous Ag-rGO (mp-AG) composite in the ETL, achieving a PCE of 20.6% (Fig. 2f) [77]. The incorporation of rGO not only improves the perovskite film quality by facilitating a larger grain size and more homogeneous morphology but also enhances charge transport because of its inherent electrical conductivity.

There is a growing demand for MOG materials that retain the crystallinity of graphite with minimal structural damage, exceed the degree of crystallinity restoration achieved by rGO, and closely approach the intrinsic properties of graphene. TCE materials must possess optical transparency comparable to that of graphene, as well as an electrical conductivity that approaches that of metals. rGO exhibits improved electrical properties compared to that of GO because of the partial removal of oxygen-containing functional groups and partial recovery of crystallinity, making it a promising candidate for use as a TCE in solar cells. However, the conductivity of rGO is still significantly lower than that of conventional metal-based electrodes, limiting its practical application as a TCE. In the case of CVD-grown graphene, which is lowly-oxidized during the fabrication process, exhibits excessively high electrical conductivity. Kang et al. fabricated Cu/CVD graphene hybrid films that functioned as both p- and n-type transparent conducting electrodes in conventional and inverted organic solar cells (Fig. 2g) [64]. The sheet resistance of these hybrid films was reduced to ~ 75 Ω sq^− 1^ with an optimal Cu thickness of 7 nm (Fig. 2h). A PSC employing a graphene/ZnO ETL, in which monolayer graphene is synthesized via low-pressure chemical vapor deposition, exhibited a nearly 60% increase in the PCE relative to that of the ZnO ETL-based PSCs (Fig. 2i) [63, 67]. This substantial enhancement is attributed to the superior electrical conductivity of monolayer graphene compared to that of rGO. These findings suggest that MOG with preserved crystallinity, such as NOGFs, can significantly outperform conventional GO or rGO in photovoltaic applications and offer significantly improved device efficiency through superior charge transport and interfacial properties.

#### Graphene quantum dots for solar cells

Recently, GQDs have gained increasing interest in the field of photovoltaics because of their unique graphene-derived crystalline structures and tunable bandgaps. GQDs synthesized via controlled oxidation exhibit a discrete and tunable bandgap without damaging the sp^2^ carbon domains of graphite or requiring additional postprocessing steps [62, 78]. These characteristics provide superior competitiveness in the field of photovoltaics. As a component of the active-layer composite, GQDs enhance light absorption through strong interactions with the host material while maintaining electrical conductivity between layers in the resultant device, improving the overall performance. These characteristics present a promising solution to the intrinsic trade-off between optical density and charge transport in organic photovoltaic systems. Further, GQDs offer a compelling alternative to conventional active-layer additives such as metal nanoparticles and inorganic semiconductor quantum dots by serving as eco-friendly materials with distinct advantages over other graphene derivatives such as GO and rGO, which lack a discrete energy gap [45, 79, 80]. Furthermore, in CVD-grown graphene, which requires additional postprocessing steps to induce a bandgap [81], GQDs can be synthesized via relatively simple and scalable methods, offering substantial advantages in terms of processability.

**Fig. 3:**
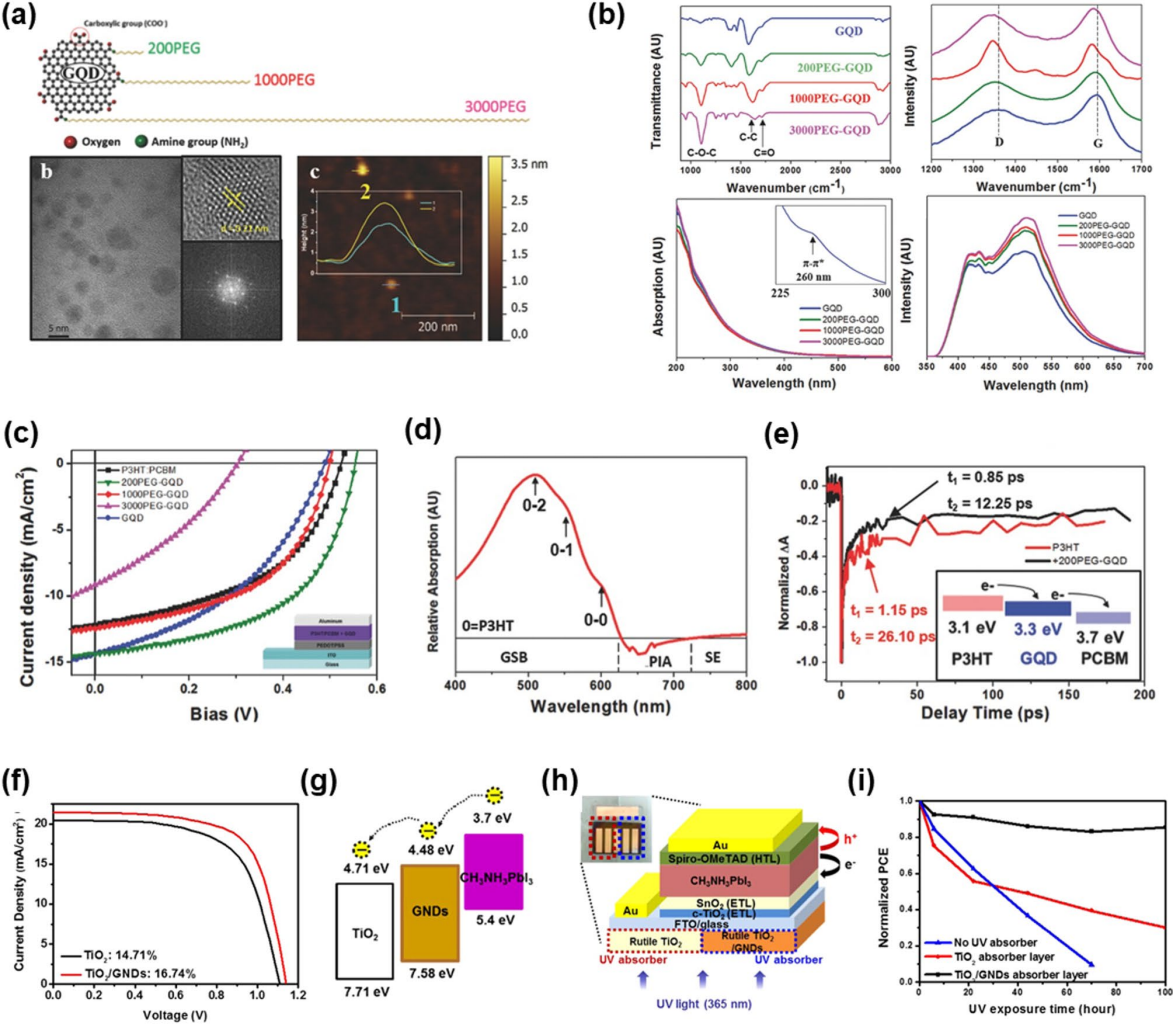
GQDs in a PV device. (**a**) Schematic and optical images of PEG-GQDs. (**b**) FT-IR spectra, Raman spectra, UV-Vis absorption, and PL intensity of GQDs and functionalized GQDs. (**c**) J–V curve of fabricated OSCs using PEG-GQDs as a component of the active layer. (**d**) Absorption of the PEG-GQDs based thin film subtracted from P3HT. (**e**) Transient absorption decay curve of the GQDs/P3HT thin film. (**a-e**) Reprinted with permission from Ref [82] (**f**) J–V curve of fabricated PSCs using GQDs as the component of ETL. (**g**) Schematic of band structure between perovskite and ETL. (**h**) Schematic of PSCs using TiO2/GQDs as an UV absorber. (**i**) PCE decay comparison of fabricated PSCs under 100 h of UV radiation. (**f-i**) Reprinted with permission from Ref [39]

Novak et al. demonstrated the enhanced photovoltaic performance of organic solar cells by incorporating PEG-functionalized graphene quantum dots (PEG-GQDs) as additives within the emitting layer; this enhancement was attributed to accelerated exciton separation facilitated by the introduction of optimally tailored PEG-GQDs structures [82]. As shown in Fig. 3a, the structures of PEG-GQDs with varying PEG chain lengths were confirmed through optical analyses. The Raman spectroscopy and FT-IR results revealed that the synthesized pristine GQDs maintained high crystallinity and exhibited relatively low levels of oxygen-containing functional groups, which suggests successful fabrication under a carefully controlled atmosphere (Fig. 3b). Although longer PEG chains led to increased photoluminescence and absorption because of the enhanced charge transfer at the GQDs edges, the J–V characteristics in Fig. 3c revealed that the shortest PEG chain achieved the highest photovoltaic efficiency. The device exhibited a PCE of 4.24%, outperforming devices incorporating longer PEG chains.

The PEG-GQDs exhibited only minor changes in absorption compared to those of pristine GQDs; however, an enhancement in absorbance was observed upon incorporation into the P3HT film because of the strong intermolecular interactions within the 500–600 nm wavelength range. As shown in Fig. 3e, the maximum increase in absorbance occurred at 509 nm, with additional shoulder peaks at ~ 550 and 600 nm (Fig. 3d). These peaks correspond to ground-state bleaching, which indicates a reduced number of bound excitons and higher population of electrons remaining in the ground state. This enhanced absorption suggests the acceleration of exciton dissociation. In addition, the transient absorption spectroscopy results depicted in Fig. 3e clearly demonstrate a faster absorption decay in the P3HT film incorporating 200PEG-GQDs than that in the pristine P3HT film. The slow decay component was reduced by more than half, providing direct evidence of significantly accelerated exciton separation dynamics.

Yoon et al. applied a TiO_2_ nanoparticle/GQDs composite as an ETL and measured the PCE of the fabricated PSCs [39]. The PSCs without GQDs exhibited a PCE of 14.71% owing to the increased UV absorption resulting from the charge transfer between the GQDs and TiO_2_, whereas those using TiO_2_/GQDs as the ETL exhibited a PCE of 16.74%, achieving an enhanced PV performance (Fig. 3f). This improvement was attributed to the discrete bandgap of GQDs. Within the rationally designed band structure of the fabricated PSCs, the highest occupied molecular orbital (HOMO) and lowest unoccupied molecular orbital (LUMO) levels of the low-oxidized GQDs were located between the valence band (VB) of the perovskite and the conduction band (CB) of TiO_2_, which facilitated efficient electron transport (Fig. 3g). Although a lower EQE was observed compared to the devices without GQDs in PSCs fabricated by applying TiO_2_/GQDs as UV absorbers (Fig. 3h), the device still showed only a 15% decrease in PCE, exhibiting exceptional performance under a 100 h UV irradiation aging test (Fig. 3i). The application of GQDs as components in PV devices effectively addresses the limitations of GO and rGO, which lack discrete and stable bandgaps, as well as those of pristine graphene, which has a gapless structure [83].

### Photothermal conversion systems

#### Mechanism of photothermal conversion and advantages of MOG

Photothermal conversion, which transforms solar radiation into localized heat, has emerged as a promising route for clean water generation, thermal catalysis, and medical therapy [84]. To achieve high conversion efficiency, photothermal materials must satisfy three critical criteria: strong and broadband solar absorption, rapid non-radiative carrier relaxation, and effective thermal conductivity for heat dissipation [85]. Graphene and its derivatives have gained attention due to their broadband optical response, high thermal conductivity, and structural tunability. In particular, NOGFs and low-oxidized GQDs shows distinct advantages in all three aspects.

Graphene exhibits strong and broadband light absorption across the ultraviolet to near-infrared (UV-NIR) spectrum due to its extended sp^2^-conjugated π-electron system and gapless band structure, which facilitate π-π^*^ transitions under a wide range of photon energies [86]. In contrast, GO contains abundant oxygen functional groups that disrupt this conjugation, localize electronic states, and open a pseudo-optical bandgap, significantly diminishing visible and NIR absorption. rGO partially restores π-conjugation, enhancing light absorption but still suffers from defect-induced losses. MOG, particularly for NOGFs produced via non-oxidative intercalation exfoliation, exhibits near-blackbody absorption (> 98%) across the solar spectrum. This structural integrity makes MOG a promising material for efficient solar energy harvesting. Low-oxidized GQDs exhibit discrete energy levels and enhanced UV absorption due to quantum confinement and surface states [39].

Efficient photothermal materials must convert absorbed photon energy into heat via non-radiative relaxation. In GO, disrupted conjugation and lattice disorder create recombination centers and trap states that impede carrier thermalization [87]. While rGO mitigates this to some extent, its structural defects still limit energy dissipation. MOG materials—particularly those derived through non-oxidative or mildly oxidative exfoliation—retain continuous π-electron networks and low defect density, enhancing electron–phonon coupling and facilitating rapid carrier relaxation. For example, the photothermal conversion efficiency (η) of graphene has been reported as ~ 67% at 808 nm, surpassing GO (~ 58%), with the efficiency of GO dropping significantly at longer wavelengths [44]. GQDs, when appropriately functionalized, also support localized energy dissipation, contributing to efficient nanoscale heat generation in composite systems [39].

Thermal transport is essential for distributing generated heat and ensuring spatially uniform photothermal effects. Single-layer pristine graphene exhibits ultrahigh in-plane thermal conductivity (~ 5000 W m^− 1^ K^− 1^), ideal for minimizing thermal gradients [88]. This property, however, is drastically reduced in GO due to sp^3^ hybridization and structural distortion. rGO recovers part of this performance but remains limited by residual defects. MOG, with its minimal oxidation and intact lattice, preserves high thermal conductivity in both standalone and composite forms. For instance, NOGFs/epoxy composites demonstrate significantly enhanced thermal conductivity (~ 1.5 W m⁻¹ K⁻¹ at 10 wt %), supporting efficient heat dissipation in solar absorber systems. While GQDs contribute less to long-range conduction, they enable localized thermal modulation when embedded in functional matrices.

Collectively, the synergistic features of MOG, which include its enhanced solar absorption, efficient light-to-heat conversion, and superior thermal conductivity, make it a highly competitive platform for advanced photothermal technologies.

#### Direct utilization of the photothermal effect with MOG

**Fig. 4:**
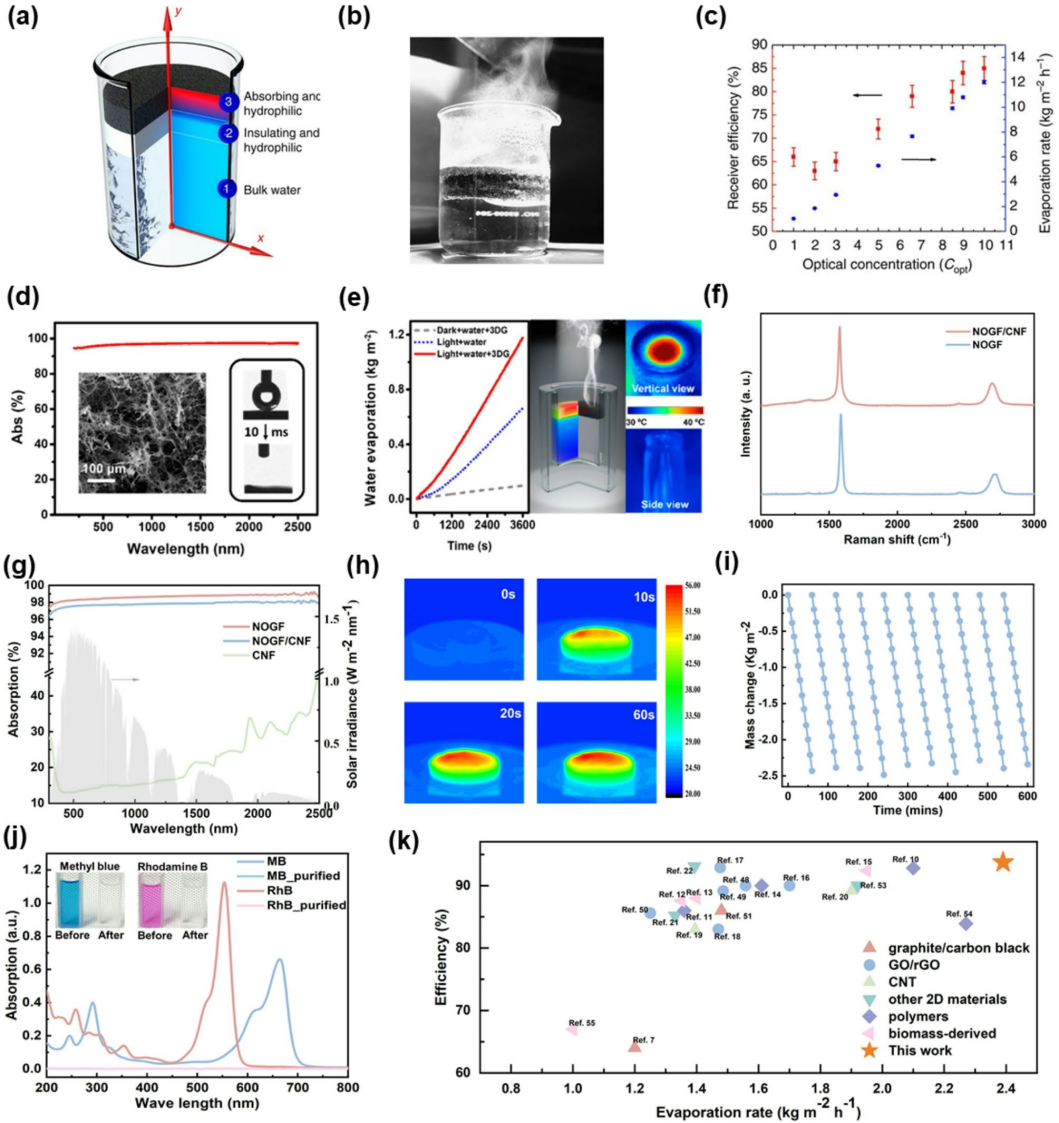
Solar steam generation performance based on exfoliated graphite, rGO, and NOGF as photothermal materials. (**a**) Representative structure for the localization of heat; the cross-section of structure and temperature distribution. (**b**) Picture of enhanced steam generation using the DLS structure under the solar illumination of 10 kW m^− 2^. (**c**) Solar thermal efficiency of the evaporation process by the DLS under a range of optical concentrations (left-hand side axis) and corresponding evaporation. (**a-c**) Reprinted with permission from Ref [91] (**d**) Absorption spectrum of 3D rGO (3DG), which shows a blackbody-like property with ~ 97% absorption across 200–2500 nm. (**e**) Left part shows the evaporation mass of water with/without 3DG under 1 kW m^–2^ solar irradiation and the case without light; the middle shows the schematic of 3DG for the vapor generation and temperature distribution (cross-section); the right part is the IR image of the vertical and side view under 1 kW m^–2^. (**d-e**) Reprinted with permission from Ref [44] (**f**) Raman spectra of NOGFs and NOGFs/CNF aerogels. (**g**) Solar absorption of CNF, NOGFs, and NOGFs/CNF aerogels. (**h**) IR images of NOGFs/CNF aerogels within 60 s. (**i**) Stability test for 10 h. (**j**) Purification of dye-contaminated water (Methyl blue and Rhodamine B). (**k**) Comparisons of evaporation rate and efficiency with the reported literatures. (**f-k**) Reprinted with permission from Ref [32]

Solar steam generation (SSG) is a promising and sustainable technology for producing clean water by harnessing sunlight. In SSG, solar energy is absorbed and converted to heat at the water–air interface, achieving efficient evaporation with minimal environmental impact [89]​. The key requirements for high-performance SSG include broad-spectrum solar absorption, efficient photothermal conversion of light to heat, effective water supply to the hot interface, and thermal localization to minimize heat loss to bulk water​. Early demonstrations showed that it was possible to localize heat and achieve high steam generation efficiencies even at moderate solar flux with an appropriate absorber structure (e.g., porous, insulating, and hydrophilic materials) ​ [90]. Figure 4a shows the schematic of a thermally localized solar absorber structure and heat transport pathway. Subsequent advances in materials and structures have made SSG a viable route for desalination and wastewater treatment without the need for costly optical concentrators or complex infrastructure [91]​.

Early pioneering work employed exfoliated graphite-based absorbers, achieving substantial advances by efficiently localizing heat at the evaporation interface and reducing heat loss compared to that with traditional bulk heating methods. For example, Ghasemi et al. demonstrated a double-layer structure (DLS) composed of exfoliated graphite supported by a porous carbon foam (Fig. 4b) [91]. This configuration achieved highly efficient heat confinement and capillary-driven water supply to the evaporation zone. Under an optical concentration of 10 kW m^− 2^, the DLS achieved a solar-to-steam conversion efficiency of 85%, significantly outperforming nanofluid and single-layer graphite absorbers​. The exfoliated graphite layer, with a surface area of 320 m^2^ g-^1^ and 97% solar absorption across 250–2250 nm, effectively created a localized hot spot while maintaining the underlying water near ambient temperature. Despite these advancements, exfoliated graphite-based materials still face critical issues, which includes a relatively high thermal conductivity that causes significant conductive heat losses, structural instability, and limited scalability because of their mechanical brittleness [92].

To address these limitations, subsequent studies extensively explored GO and rGO as alternative absorbers. Their advantages include scalable fabrication, strong hydrophilicity, and broad solar absorption. For example, Zhang et al. developed a vertically aligned graphene sheet membrane (VA-GSM) fabricated from GO using antifreeze-assisted directional freezing, which enabled efficient capillary-driven water transport and solar absorption [93]. The composite achieved a solar-thermal efficiency of 86.5% under 1 sun and 94.2% under 4 sun, with an evaporation rate of 6.25 kg m^− 2^ h^− 1^, demonstrating the advantages of directional structure and water management​. Based on these features, rGO-based systems further enhanced light absorption, thermal confinement, and material stability, which improve the evaporation rate and photothermal conversion efficiency further. Yang et al. fabricated a self-supporting 3D graphene foam via solvothermal reduction, yielding a porous, cross-linked rGO network [94]. Compared to GO structures, the rGO foam exhibited stronger broadband absorption—exceeding 94% across 250–2500 nm, owing to its higher graphitization and reduced light scattering (Fig. 4d). The foam achieved a solar-to-vapor efficiency of 87% under 1 sun, and enabled stable interfacial heating and vapor generation, as confirmed by IR imaging and water evaporation measurement (Fig. 4e)​. While GO and rGO significantly outperform bulk graphite by enabling interfacial heating and structural tunability, their photothermal response is still limited by high defect densities and limited solar absorption. The presence of oxygen-containing groups also compromises long-term thermal stability and heat transport.

To further improve photothermal efficiency, NOGFs have been developed, offering excellent broadband solar absorption and superior thermal stability. Chen et al. fabricated a tree-inspired aerogel comprising NOGFs and cellulose nanofibers (CNFs) via a bidirectional freeze-casting technique [32]. Raman spectra confirmed the high quality of NOGFs, exhibiting a minimal defect density with an ID/IG ratio of only ~ 0.065 (Fig. 4f). After composite fabricated, the slight increase in ID/IG for NOGFs/CNF still indicated excellent sp^2^-lattice preservation. The aerogel achieved outstanding broadband absorption, exceeding 97% across the 250–2500 nm range for both NOGFs and NOGFs/CNF samples, as shown in Fig. 4g. This absorption was significantly higher than typical GO/rGO-based systems, ensuring efficient solar energy harvesting. Thermal imaging under 1 sun illumination (Fig. 4h) demonstrated rapid and localized heating at the evaporation interface within 60 s, confirming strong light-to-heat conversion confined to the surface. The hierarchical structure, characterized by radially aligned microchannels, facilitated rapid capillary-driven water transport and minimized heat loss. Consequently, the aerogel achieved a stable water evaporation rate of 2.39 kg m^− 2^h^− 1^ and an energy conversion efficiency of 93.7% under 1 sun, among the highest values reported for carbon-based materials. Long-term cycling tests further showed excellent mass loss linearity without performance degradation due to the minimum of defects in NOGFs. Beyond high evaporation performance, the NOGFs/CNF aerogel also demonstrated effective water purification capabilities. As shown in Fig. 4j, the system efficiently removed organic dyes such as methylene blue and rhodamine B after photothermal treatment. In a broader performance comparison (Fig. 4k), this work outperformed conventional graphite/carbon black, GO/rGO, CNT, and biomass-derived absorbers, setting a new benchmark for combined evaporation rate and efficiency. These results proved the exceptional potential of structurally intact, high-absorption NOGFs-based aerogels for sustainable, high-efficiency solar-driven water purification and desalination technologies.

Beyond water evaporation, the exceptional photothermal properties of NOGFs also enable their application in areas such as anti-icing, de-icing, and photothermal disinfection. Their high broadband absorption (> 98%), outstanding thermal conductivity, and structural integrity make them particularly effective under extreme or harsh environmental conditions. For instance, Xie et al. incorporated graphene nanosheets into polymer matrices to fabricate durable photothermal de-icing surfaces [95]. The resulting polyethylene/graphene composite exhibited superhydrophobicity, ultralow reflectance (~ 1.6%), and high mechanical and chemical robustness, enabling efficient photothermal de-icing under solar illumination. Similarly, Pei et al.​ developed polypropylene/graphene foams with optimized pore structures, achieving rapid surface heating to ~ 94 °C within 5 min and complete de-icing within 70 s under natural sunlight [96]. In the biomedical field, Zhong et al.​ applied a laser-induced graphene with low defects coating onto commercial surgical masks, showing superhydrophobicity and photothermal sterilization capability [97]. Upon sunlight exposure, the graphene-coated mask surface rapidly heated above 80 °C, enabling effective self-cleaning and potential for repeated safe use. These findings demonstrate the broad applicability of NOGFs as high-performance photothermal materials, supporting their integration into diverse fields ranging from water purification to environmental and healthcare technologies.

#### Photothermal-assisted thermal storage and energy conversion with MOG

Photothermal-assisted thermal storage and energy conversion technologies have rapidly evolved, offering promising strategies for sustainable energy harvesting and utilization [98]. Compared to conventional GO or rGO, MOG combines superior structural integrity, outstanding photothermal conversion efficiency, and optimized thermal management capabilities, making it suitable for high-performance energy systems.

**Fig. 5:**
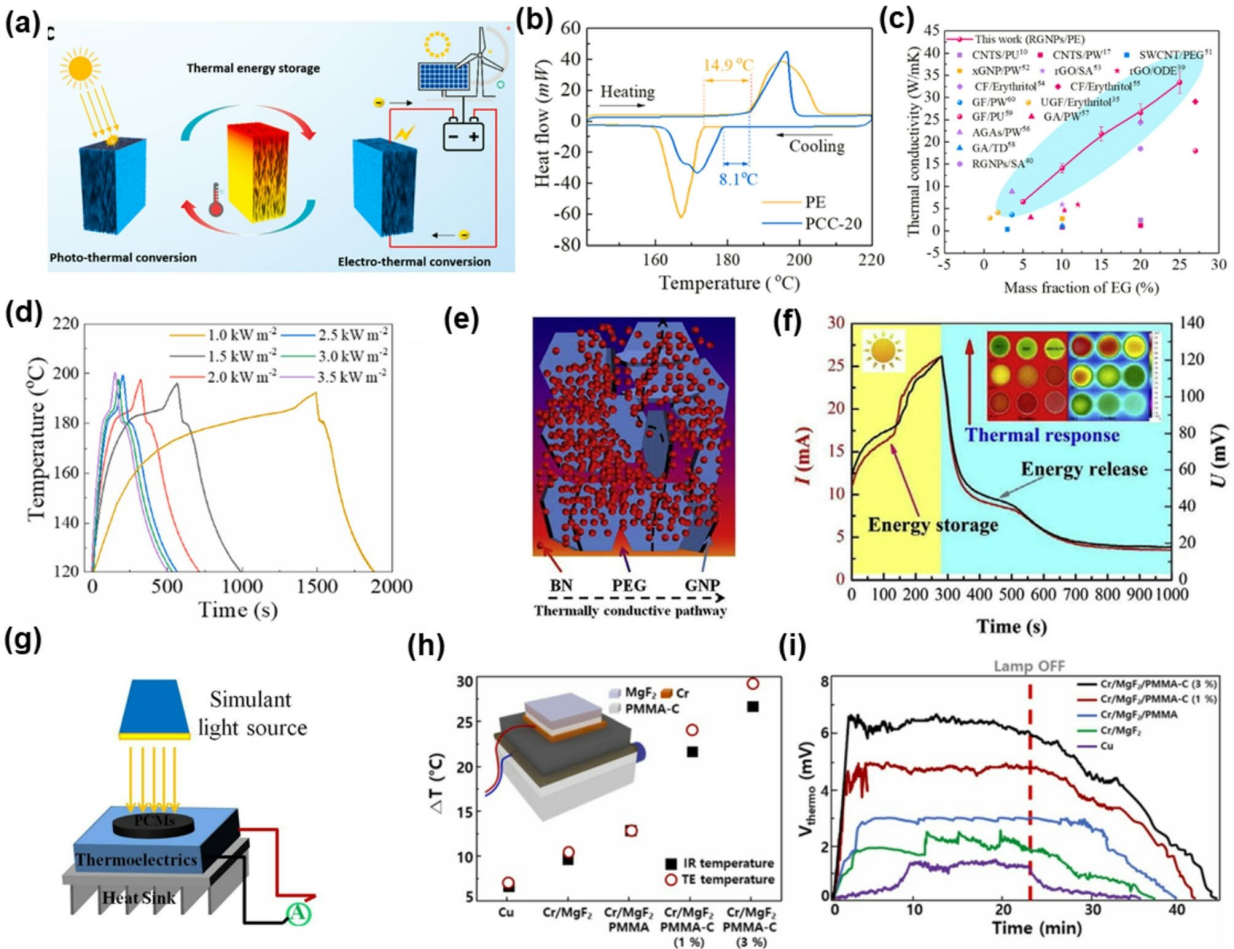
Graphene flakes for solar-assisted energy conversion and storage systems. (**a**) Thermal design of versatile PCCs for photo/electro-thermal energy harvesting and storage. (**b**) Differential scanning calorimetric (DSC) curves of pristine PE and PCC-20 during the heating and cooling processes. (**c**) Comparison in terms of thermal conductivities between our PCCs and the reported carbon-based PCCs. (**d**) Time-temperature evolution curves of the PCC-based integrated solar photo-thermal energy device at different solar irradiations. (**a-d**) Reprinted with permission from Ref [99] (**e**) Schematic of thermally conductive pathway in the 30BN/1GNP composite PCMs. (**f**) I–t and U–t curves of 30BN/1GNP under sunlight radiation. (**g**) Experimental setup for light-to-electric conversion. (**e-g**) Reprinted with permission from Ref [100] (**h**) Comparison of ∆T values found using the IR camera and calculated using the output voltage of the TEG. (**i**) Output voltage of the hybrid photothermal structure generated by TEG. (**h-i**) Reprinted with permission from Ref [101]

Recent advances have demonstrated that integrating MOG materials into phase change composites and thermoelectric systems enables more efficient solar-thermal energy storage and conversion. Li et al.​ developed highly conductive phase change composites (PCCs) by integrating vertically aligned reticulated graphite nanoplatelets (RGNPs) into a polyethylene matrix [99]. A dual-mode energy storage system was constructed, where solar energy is first converted into thermal energy (photo-thermal conversion) and subsequently into electricity via electro-thermal conversion as shown in Fig. 5a. The fabricated materials, retaining partially preserved van der Waals interactions and substantial sp^2^ domains, exhibited anisotropic thermal conductivity pathways. Differential scanning calorimetry (DSC) measurements revealed improved phase change behavior, with a broader heating window and narrower cooling range for the RGNP-enhanced composite (Fig. 5b). The in-plane thermal conductivity reached 33.5 W m^− 1^K^− 1^ at 20 wt% RGNP loading, as measured by the laser flash method, which significantly exceeds that of conventional carbon-based PCCs (Fig. 5c). Under concentrated sunlight, the PCC surfaces achieved temperatures exceeding 186 °C, enabling efficient solar thermal storage without significant heat loss (Fig. 5d). While graphite-based architectures improve bulk thermal transport, NOGFs can further optimize interfacial heat transfer and light absorption, enabling enhanced performance even at low loading levels. Yang et al.​ incorporated a small amount (1 wt %) of non-oxidized graphene nanoplatelets (GNPs) into poly(ethylene glycol)/boron nitride (PEG/BN) composite phase change materials to further enhance their thermal and photothermal performance [100]. As illustrated in Fig. 5e, the introduction of GNPs formed efficient thermally conductive networks within the PEG matrix, bridging boron nitride (BN) particles and significantly reducing the thermal resistance. The improved thermal conductivity directly contributed to significantly enhanced photothermal energy storage performance. Under solar illumination at 2.5 kW m^− 2^, the PEG/BN/GNP composite exhibited a rapid temperature rise, reaching approximately 196 °C within 360 s, significantly improved compared with the PEG/BN composite without GNPs (Fig. 5f).

Beyond thermal storage, MOG materials have also been explored for enhancing photothermal-thermoelectric energy conversion, where efficient heat harvesting and retention are critical to maintaining large temperature gradients across thermoelectric devices. Kwak et al.​ reported a hybrid photothermal structure integrating a Cr-MgF_2_ multilayer solar absorber with a PMMA-graphene composite heat reservoir (Fig. 5g) [101]. The inclusion of graphene significantly boosted the thermal conductivity of the reservoir layer by ~ 47%, facilitating effective heat storage and transfer. As shown in Fig. 5h, the hybrid structure, achieved a sustained temperature difference (ΔT) of ~ 27 °C across the thermoelectric generator (TEG), compared to less than 10 °C for the Cu reference system. This enhanced thermal gradient led to a 2.74-fold increase in the thermoelectric output voltage, reaching a maximum of ~ 140 mV under continuous illumination (Fig. 5i). The output remained stable throughout the operation period and declined sharply only after the light source was turned off, demonstrating the system’s effective photothermal-thermoelectric coupling.

In addition to energy storage and thermoelectric conversion, photothermal effects can significantly enhance catalytic reactions by elevating local temperatures and facilitating charge transfer, making MOG materials promising candidates for photothermal-assisted catalysis. For example, Wang et al.​ fabricated a graphene-MnO_2_ nanohybrid, where graphene effectively transferred solar thermal energy to MnO_2_, enhancing its surface temperature significantly and promoting formaldehyde oxidation [102]. Under Xenon lamp irradiation, the MnO_2_-graphene composite achieved an approximately 80% transformation rate of HCHO to CO_2_, significantly higher than pristine MnO_2_. This enhancement was attributed to the synergistic photothermal effect and interfacial charge transfer between graphene and MnO_2_. These results demonstrate the potential of MOG materials to extend their photothermal advantages into catalytic reactions. Detailed catalytic mechanisms and broader applications will be discussed in the photocatalysis section, while this part focuses on the photothermal utilization aspect.

Recently, GQDs have also been employed in photothermal-assisted energy storage systems, offering unique advantages due to their tunable electronic structure, strong light absorption, and excellent interfacial compatibility. Chang et al.​ developed a 3D-printed biomimetic supercapacitor integrating GQDs with MXene, inspired by spiral grass structures to enhance solar absorption and thermal confinement [103]. Under 1 sun illumination, the device surface reached 67.6 °C, and the areal capacitance increased by 3.04 times compared to the dark condition. The hybrid system delivered a high energy density of 1.18 mWh cm^− 2^ and retained 94.1% of its capacitance after 10,000 cycles in the dark, demonstrating robust long-term stability. The enhanced performance was primarily attributed to GQDs, which acted as photoresponsive nanoscale spacers that improved light harvesting and promoted efficient ion/electron transport at the MXene interface. This work demonstrates the potential of GQDs as multifunctional photothermal additives for hybrid energy storage systems under solar input.

In parallel, low-oxidized GQDs offer unique advantages for nanoscale photothermal applications. Their quantum confinement effects enable tunable bandgaps (2.5–4.0 eV), allowing precise absorption in the UV-vis spectrum for targeted energy conversion [24, 33–37]. The abundant edge functional groups (–OH,–COOH) facilitate interfacial coupling with polymers or semiconductors, enhancing localized heat generation via non-radiative relaxation [39, 103]. Unlike NOGFs, GQDs achieve efficient photothermal conversion at ultralow concentrations in composite systems, minimizing material usage while maintaining high conversion efficiency (> 80%) under solar irradiation [103]. These properties make GQDs ideal for biomedical photothermal therapy, solar-driven microreactors, and precision thermal management applications where nanoscale control is critical.

Overall, MOG emerges as a promising material platform across various photothermal-assisted applications due to its unique combination of high photothermal conversion efficiency, excellent thermal management capabilities, structural integrity, and long-term stability. Ongoing advances in MOG -based systems are expected to push the boundaries of solar energy utilization, unlocking new possibilities for integrated and sustainable energy technologies.

### Photocatalytic systems

#### Mechanism of photocatalysis and roles of MOG

**Fig. 6:**
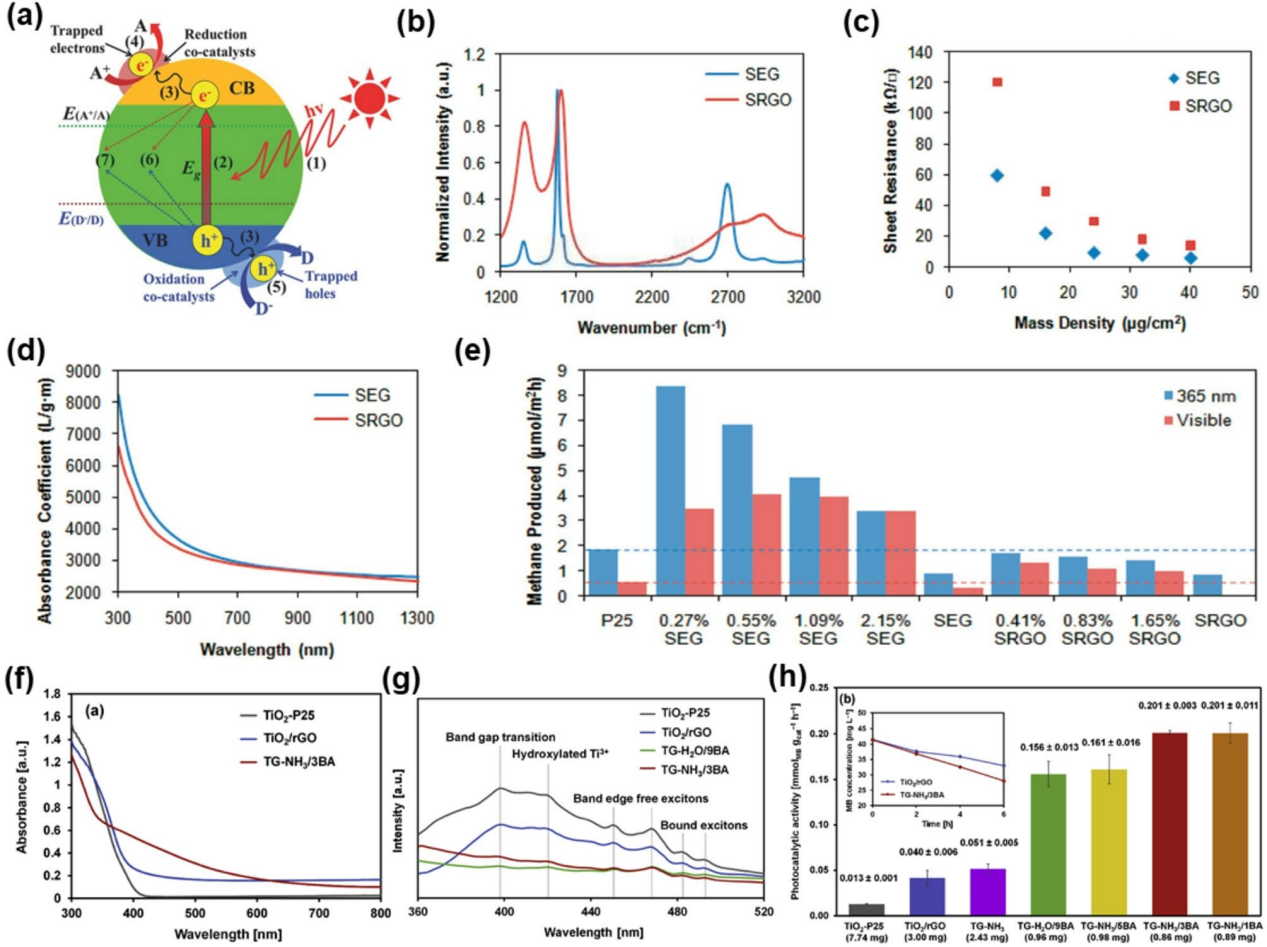
Comparison of GO, rGO, and graphene for photocatalysis. (**a**) Fundamental kinetics requirements of heterogeneous photocatalysis. Reprinted with permission from Ref [105] (**b**) Intensity-normalized Raman spectra of SEG and SRGO films annealed at 400 °C for 30 min in air. (**c**) Sheet resistances of SEG and SRGO thin films formed via vacuum filtration as a function of mass density. (**d**) Optical absorbance coefficients for SEG and SRGO dispersions in DMF. (**e**) CO_2_ photoreduction for SEG–P25 and SRGO–P25 nanocomposites under ultraviolet (365 nm) and visible illumination. (**b-e**) Reprinted with permission from Ref [106] (**f**) UV–Vis DRS spectra of TiO_2_-P25, TiO_2_/rGO, and TiO_2_/graphene nanocomposites. (**g**) PL spectra of TiO_2_-P25, TiO_2_/rGO, and TiO_2_/graphene nanocomposites. The excitation source of 300 nm was employed. (**h**) Photocatalytic activity per gram catalyst. (**f-h**) Reprinted with permission from Ref [107]

Photocatalysis is a promising sustainable technology for environmental remediation and energy production that operates through semiconductor-based heterogeneous catalysts [104]. Illumination with a light of energy greater than or equal to the bandgap of the semiconductor excites electrons from the VB to the CB, leaving behind positively charged holes in the VB. These photogenerated electron–hole pairs are crucial for initiating redox reactions. Electrons in the CB typically participate in reduction reactions, whereas holes in the VB facilitate oxidation reactions (Fig. 6a). Several factors affect the efficiency of photocatalysis, including the semiconductor surface properties, availability of catalytic active sites, and interface interactions between the photocatalyst and reactants. The rapid recombination of photogenerated charge carriers remains a significant challenge, severely limiting the quantum efficiency and overall catalytic performance [105].

Graphene has emerged as a significant candidate for enhancing photocatalytic efficiency because of excellent electronic conductivity, large specific surface area, and robust chemical stability. Graphene functions as an electron mediator, effectively suppressing charge recombination by accelerating charge separation and transport [108]. In addition, the large surface area of graphene increases its adsorption capacity, which facilitates stronger interactions with the reactants. Further, they form intimate heterojunctions with semiconductor photocatalysts, creating internal electric fields that enhance electron transfer and extend charge carrier lifetimes, significantly improving the photocatalytic performance. GO is a heavily oxidized derivative of graphene that has been widely studied because of its superior dispersibility in aqueous media, abundant oxygen-containing functional groups, and ease of chemical modification. These functional groups significantly enhance interfacial interactions between GO and semiconductor materials, facilitating effective electron transfer across the interface [109]. However, the poor electrical conductivity severely limits its performance as a photocatalytic co-catalyst [110]. In contrast, MOG presents a more balanced approach by carefully tuning the oxidation levels, preserving significant sp^2^ carbon domains, and simultaneously introducing a moderate number of functional groups. This strategic approach maintains high conductivity and efficient electron transport while improving hydrophilicity and semiconductor interface compatibility. MOG effectively mitigates electron–hole recombination through enhanced charge separation, providing improved catalytic efficiency and greater stability under photocatalytic conditions than heavily oxidized GO.

GQDs extend the advantages of MOG and introduce unique quantum confinement effects and customizable electronic properties that are highly beneficial for photocatalytic applications. Low-oxidized GQDs fabricated via GIC-based nondestructive exfoliation retain substantial sp^2^ subdomains along with abundant surface functional groups, ensuring excellent electron mobility, abundant catalytic active sites, and superior interfacial coupling with semiconductor photocatalysts. These combined features allow GQDs to significantly enhance visible light absorption, improve electron–hole pair separation efficiency, and offer tailored interactions with reactants.

Thus, the strategic control of graphene oxidation states (from GO through MOG to GQDs) represents a powerful approach toward addressing conventional photocatalytic limitations. Optimizing the electronic properties of graphene and its interfacial chemistry is crucial for developing next-generation photocatalytic systems with significantly improved environmental and energy-related performances.

#### Performance comparison of graphene-based photocatalysts

Graphene and its derivatives have gained extensive attention as essential components in photocatalytic systems, primarily due to their excellent electrical conductivity, large specific surface area, and unique two-dimensional structure, facilitating effective electron transport, charge separation, and surface reactions. Although GO is widely used in photocatalysis due to its excellent aqueous dispersibility, its limited conductivity impairs electron mobility, thereby hindering overall photocatalytic efficiency [111, 112].

​ To overcome the limitations of GO, rGO has been employed as a more conductive co-catalyst. Partial restoration of the sp^2^ carbon framework enhances electron transport and suppresses charge recombination. Zou et al. reported that Cu_2_O-rGO composites achieved a first-order rate constant of 0.651 h^− 1^ for methylene blue degradation under visible light, significantly higher than the 0.185 h^− 1^ observed for Cu_2_O alone​ [113]. The enhanced performance was attributed to improved visible-light absorption, suppressed photogenerated charge recombination as evidenced by reduced PL intensity, and higher generation of superoxide radicals. To further improve the charge transfer efficiency of rGO-based photocatalysts, incorporation of highly conductive graphene has been demonstrated as a powerful strategy. Lu et al. constructed a 3D rGO aerogel hybridized with 15 wt % commercial graphene (EGR), which exhibited a significantly enhanced electrical conductivity and interfacial charge separation compared to pristine rGO aerogel [114]. This hybrid aerogel achieved nearly complete reduction of Cr(VI) under visible light within 20 min, while the rGO-only aerogel showed much lower activity under identical conditions. The improvement was attributed to the superior electron mobility of EGR and its role as both an electron sink and a conductive bridge, facilitating charge separation and transfer across the hybrid framework​. Similarly, Hu et al. incorporated 1 wt % graphene into a TiO_2_-based photocatalytic Janus membrane [115]. This modification enhanced the CH_4_ yield during CO_2_ photoreduction from 8.9 ppm (for pure P25 system) to 11.1 ppm. The added graphene extended charge carrier lifetimes by suppressing electron-hole recombination and increased the probability of photogenerated electrons reacting with CO_2_, without compromising light absorption. However, excessive graphene (10 wt %) led to a performance drop due to surface shielding effects, highlighting the importance of optimal graphene loading​. These studies collectively prove the critical role of graphene-based architectures in improving photocatalytic performance and necessitate the need for oxidation-controlled graphene materials that simultaneously maintain high conductivity and interfacial compatibility.

**Fig. 7:**
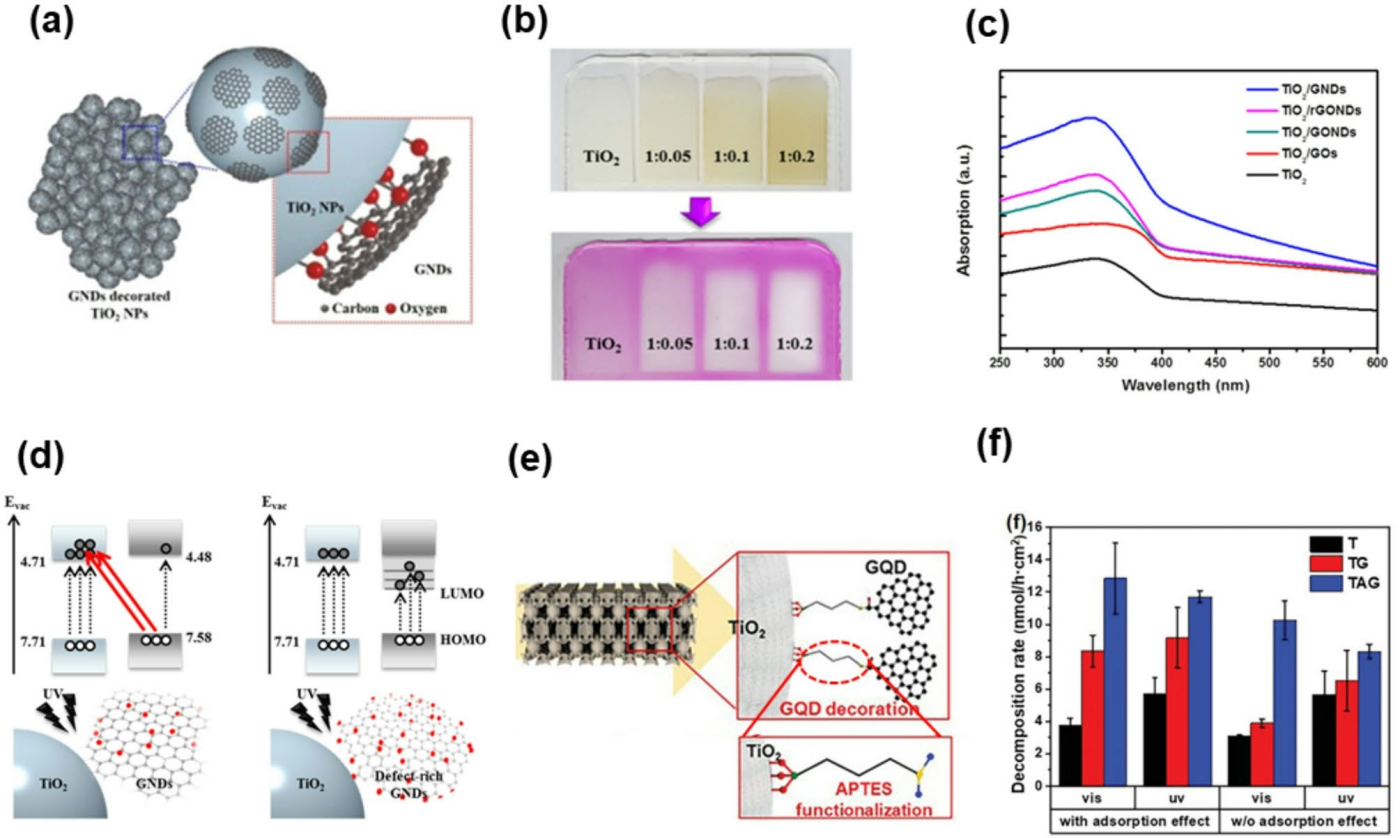
Low-oxidized GQDs for photocatalysis. (**a**) Schematic of TiO_2_ NP/GQDs composites. (**b**) Images demonstrating the UV-blocking effect of the corresponding TiO_2_ NP/GQDs composites before (top) and after (bottom) UV irradiation at 365 nm. (**c**) UV/vis absorption spectra of TiO_2_ NPs incorporated with various graphitic materials. (**d**) Schematic illustrations of the charge-transfer process occurring between TiO_2_ NPs and GQDs of low (left) and high (right) defect densities. Reprinted with permission from Ref [39] (**e**) Schematic of low oxidized GQDs decorated monolithic anatase 3D titania via (3-aminopropyl)triethoxysilane linker. (**f**) MB decomposition rate including adsorption effect and except adsorption effect of T, TG, and TAG under UV and visible irradiation. (**e-f**) Reprinted with permission from Ref [117]

MOG, especially NOGFs, has emerged as a superior co-catalyst in photocatalysis by integrating high electrical conductivity with preserved π-conjugation and minimal defect density. Liang et al. systematically compared solvent-exfoliated graphene (SEG) with solvent-rGO (SRGO) in TiO_2_ nanocomposites and highlighted key structure–property–performance correlations across several metrics [106]​. As shown in Fig. 6b, Raman spectra shows a much lower ID/IG ratio (0.17) for SEG compared to SRGO (0.82), indicating significantly fewer defects. This structural integrity directly impacts electronic transport, as SEG films exhibit up to 2.4-fold lower sheet resistance than SRGO at similar mass densities (Fig. 6c), confirming superior carrier mobility. Optical absorbance spectra (Fig. 6d) show that SEG maintains higher absorbance coefficients across the UV–vis–NIR region, beneficial for light harvesting. These structural and electronic advantages contribute to the significantly improved photocatalytic performance. In CO_2_ photoreduction (Fig. 7e), TiO_2_-SEG composites generated up to 8.6 µmol g^− 1^ h^− 1^ of CH_4_ under UV light (0.27 wt % SEG), a 7.2-fold increase over pure TiO_2_ and significantly higher than all SRGO-based composites. Moreover, Ton et al. synthesized TiO_2_/NOGFs composites (TG-NH_3_/3BA) via a one-pot chemical exfoliation strategy, enabling intimate integration of high-quality graphene with anatase TiO_2_ [107]​. As shown in Fig. 7f, the UV-Vis spectra reveal that TG-NH_3_/3BA exhibits significantly enhanced visible-light absorption, with an extended absorption edge beyond 570 nm, compared to TiO_2_-P25 and TiO_2_/rGO. In Fig. 7g, photoluminescence spectra demonstrate strong emission quenching for the NOGFs composite, indicating efficient suppression of charge recombination. These optical advantages result in outstanding photocatalytic performance. As shown in Fig. 7h, the TG-NH_3_/3BA composite achieved a visible-light methylene blue degradation rate of 0.201 mmol g^− 1^ h^− 1^, much higher than TiO_2_/rGO (0.041) and TiO_2_-P25 (0.013). Additionally, Garrison et al. demonstrated that conformally coating ammonium perchlorate (AP) crystals with NOGFs significantly accelerated their thermal decomposition compared to both uncoated and hBN-coated AP [116]. The NOGFs-coated AP exhibited a reduced onset decomposition temperature (280 °C vs. 308 °C for pure AP) and a 500-fold increase in the Arrhenius pre-exponential factor, attributed to enhanced electron transfer across the interface​.

Thus, MOG strategically combines the beneficial attributes of GO and pristine graphene, which makes it highly promising for future developments in photocatalysis, including pollutant degradation, CO_2_ conversion, and renewable energy generation.

#### Graphene quantum dots for photocatalysis

GQDs, including both graphene oxide quantum dots (GOQDs) and low-oxidized GQDs, are nanoscale carbon materials typically less than 10 nm in size. Their nano-size facilitate uniform dispersion and intimate contact with semiconductor surfaces, such as TiO_2_ and ZnO, enhancing interfacial charge transfer [118]. A key advantage of GQDs is their size-dependent bandgap, arising from quantum confinement and edge effects, which allows for tunable optical absorption and efficient energy level alignment with various photocatalysts [119].

GOQDs are commonly produced by oxidative cutting of graphite or graphene oxide, a process that introduces abundant structural defects and oxygen-containing functional groups. They have been widely employed in photocatalysis, particularly in visible-light-driven systems. For example, Yeh et al. reported that nitrogen-doped GOQDs (NGO-QDs) could independently drive overall water splitting under visible light without any noble metal cocatalyst [120]. The NGO-QDs exhibited coexisting p- and n-type semiconducting behavior and generated a direct bandgap of 2.2 eV, with conduction and valence band edges well-aligned to water redox potentials. Xu et al. integrated GOQDs with g-C_3_N_4_, where the GOQDs acted as electron acceptors to promote charge separation, resulting in 3.1-fold higher dye degradation and 99.6% bacterial inactivation under visible light compared to pristine g- C_3_N_4_ [121]​. In another work, Gliniak et al. demonstrated that sulfur-doped GOQDs enhanced light absorption and catalytic surface reactivity, enabling hydrogen evolution rates of 303 mmol h^− 1^g^− 1^ in water and over 30,000 mmol h^− 1^g^− 1^ in ethanol under sunlight [122]. However, the high oxidation level in GOQDs disrupts π-conjugation and reduces conductivity, limiting long-range electron transport and charge separation efficiency. Their structural instability and surface disorder further compromise interfacial coupling and photocatalytic durability.

Accordingly, low-oxidized GQDs have been developed to retain sp² subdomain and improve electronic conductivity, while still maintaining sufficient surface compatibility for effective integration with semiconductor photocatalysts. A notable demonstration was reported by Yoon et al., where GQDs synthesized via a GIC-based method were integrated with TiO_2_ nanoparticles to form core-shell nanocomposites (Fig. 7a) [39]​. The resulting TiO_2_/GQDs composites exhibited a significant enhancement in UV absorption, with a molar extinction coefficient at 336 nm reaching 371.2 M^− 1^ cm^− 1^, a 2.43-fold increase compared to pristine TiO_2_ (Fig. 7c). This optical enhancement was visually evident under 365 nm UV irradiation, where the GQDs-loaded samples maintained color stability due to stronger UV shielding (Fig. 7b). The improved absorption was attributed to efficient ligand-to-metal charge transfer from the LUMO of GQDs to the TiO_2_ conduction band, facilitated by their discrete quantum-confined bandgap and preserved sp^2^ conjugation (Fig. 7d). Although direct photocatalytic degradation was not assessed in this study, the same TiO_2_/GQDs composites demonstrated a PCE of 16.74% and enhanced UV durability in photovoltaic applications, highlighting their optoelectronic potential and stability.

To apply this improvement into practical photocatalysis, they further constructed a 3D monolithic TiO_2_/GQDs heterostructure using (3-aminopropyl)triethoxysilane (APTES) as a molecular linker (Fig. 7e) [117]​. This design promoted stable interfacial bonding while suppressing back-electron transfer. As shown in Fig. 7f, the resulting composite achieved a 242% increase in methylene blue degradation under visible light and 104% under UV irradiation, even after decoupling the adsorption effect. These results underscore the role of low-oxidized GQDs not only in enhancing light absorption but also in enabling efficient charge separation through tailored energy alignment and interfacial engineering.

Low-oxidized GQDs, with their balanced structure of preserved sp² domains and moderate surface functionality, offer an effective platform for enhancing charge transport and semiconductor interface integration. Moving forward, their tunable energy levels, high dispersibility, and strong quantum confinement make them ideal for constructing tailored heterojunctions, extending visible-light activity, and enabling selective photocatalytic transformations in complex environments.

## Conclusions and outlook

MOG, including NOGFs and GQDs, preserve the sp^2^ network, offer tunable interfacial properties, and enable efficient solar energy conversion. In photovoltaic applications, NOGFs serve as promising flexible transparent electrodes with high conductivity (> 10^3^ S cm^− 1^) and transmittance (> 90%) while functioning as effective charge transport layers. GQDs enhance light harvesting through quantum confinement effects. For photothermal conversion, NOGFs exhibit exceptional anisotropic thermal conductivity (> 1000 W m^− 1^ K^− 1^ in-plane) and near-full-spectrum solar absorption (> 98%), thereby making them ideal for solar steam generation. GQDs further improve photothermal efficiency via localized surface plasmon resonance, showing great promise for biomedical applications. In photocatalysis, GQDs promote charge separation through abundant edge-active sites while NOGFs act as superior catalyst supports because of their large surface area and excellent electron mobility. Both materials can be synthesized via environment-friendly methods such as intercalation-assisted exfoliation, ensuring scalability for practical applications. These properties suggest that MOG is a promising and sustainable material for next-generation high-efficiency multifunctional solar energy technologies. To translate the promise of MOG into real-world applications, future research should focus on controllable synthesis, atomic-scale structural-function insight, and device-level integration.

One of the foremost challenges lies in the precise control of oxygen content and defect distribution at the atomic level, which is critical for tuning optoelectronic properties without compromising structural integrity. Developing in situ monitoring techniques, such as real-time Raman spectroscopy or electrochemical impedance tracking during exfoliation, will help regulate oxidation states while preserving the sp^2^-hybridized carbon framework [40, 123].

Scalability is equally critical. Among existing methods, electrochemical exfoliation, which uses voltage-controlled ion intercalation and gas evolution in aqueous media, offers a sustainable and tunable approach for large-scale MOG production. Hybrid strategies, such as plasma-assisted exfoliation, may further reduce energy consumption while maintaining structural quality. For industrial application, reproducibility and material uniformity will be important, especifically achieving narrow flake thickness distributions (± 1 nm), lateral sizes (± 20%), and controllable oxygen contents (3–15 at%) is essential for reliable device performance [124, 125].

Understanding structure-property relationships at the nanoscale remains another priority. In particular, the distinct roles of basal-plane versus edge oxidation on charge transport and catalytic activity require deeper investigation. Operando techniques, such as environmental TEM combined with electron energy loss spectroscopy (EELS), could reveal how oxygen functionalities evolve under working conditions [126]. Meanwhile, integrating DFT simulations with kinetic Monte Carlo methods may shed light on carrier dynamics across different oxidation states. As data accumulate, machine learning models trained on structural and performance datasets could accelerate the identification of optimal oxidation configurations for specific solar energy applications [127].

While MOG materials exhibit strong intrinsic properties, several practical challenges remain, particularly regarding their processability and compatibility with diverse fabrication environments. One key issue is the limited dispersibility NOGFs and GQDs in common solvents. NOGFs, with their minimal defect density and hydrophobic surfaces, are generally not dispersible in aqueous media, hindering their integration into solution-based systems. In contrast, GQDs possess abundant oxygen-containing functional groups at their edges, enabling dispersion in water but limiting compatibility with nonpolar or organic solvents. These limitations pose barriers to material incorporation, interface formation, and scalable device fabrication. To address these challenges, proper edge functionalization strategies, including covalent and non-covalent functionalization, are needed to tailor the surface chemistry of MOG materials and expand their dispersibility across a broader range of solvents [128]. Achieving stable dispersion in water, alcohols, and organic media will be essential for enabling uniform film deposition, heterostructure assembly, and hybrid device integration.

Due to their unique combination of structural integrity and functional tunability, MOG materials are increasingly being considered for integration into practical devices. For instance, flexible solar cells using NOGFs-based transparent electrodes have demonstrated excellent conductivity and optical transmittance, offering a compelling alternative to brittle ITO in emerging perovskite and organic photovoltaic platforms. Likewise, solar-driven water purification systems employing NOGFs aerogels or GQDs-coated membranes have shown high evaporation rates and effective microbial removal, pointing toward lightweight, off-grid water treatment solutions. These developments highlight the need to address key engineering challenges, such as interfacial adhesion, heat management, and large-area processability, to facilitate the transition of MOG from materials research to device implementation. Furthermore, the long-term stability of these materials under real-world stresses like UV exposure, moisture, and temperature fluctuations remains insufficiently studied, and will be essential for their broader adoption.

MOG offers a unique combination of tunable electronic structure, scalable synthesis potential, and cross-functional integration capabilities, positioning it as a promising material for next-generation solar energy systems. Realizing its full potential will require interdisciplinary efforts spanning materials design, device engineering, computational modeling, and environmental evaluation. As solar technologies evolve toward modular and multifunctional architectures, MOG is particularly well-suited to unify diverse applications, including photovoltaics, photothermal conversion, and photocatalysis, through its intrinsic electronic, optical, and catalytic properties.

## Data Availability

No new data were generated in this study. All relevant information has been obtained from published sources, which are cited in the manuscript.
